# The pregnancy outcomes among women receiving individualized algorithm dosing with follitropin delta: a systematic review of randomized controlled trials

**DOI:** 10.1007/s10815-024-03146-1

**Published:** 2024-05-29

**Authors:** Bogdan Doroftei, Ovidiu-Dumitru Ilie, Ana-Maria Dabuleanu, Theodora Armeanu, Radu Maftei

**Affiliations:** 1 Department of Mother and Child, Faculty of Medicine, University of Medicine and Pharmacy “Grigore T. Popa”, University Street No. 16, 700115 Iasi, Romania; 2 Clinical Hospital of Obstetrics and Gynecology “Cuza Voda”, Cuza Voda Street No. 34, 700038 Iasi, Romania; 3 Origyn Fertility Center, Palace Street No. 3C, 700032 Iasi, Romania

**Keywords:** Follitropin delta, FE 999049, Rekovelle, Ovarian stimulation, Pregnancy, IVF, ICSI, Infertility

## Abstract

**Purpose:**

To investigate whether the ovarian stimulation with follitropin delta in an individualized algorithm-based manner is inferior to recombinant human-follicle stimulating’s follitropin alfa or follitropin beta conventional dosing regarding a series of established primary endpoints.

**Methods:**

We conducted a registered systematic review (CRD42024512792) on PubMed-MEDLINE, Web of Science™, Cochrane Database of Systematic Reviews, and Scopus. Our search was designed to cover all relevant literature, particularly randomized controlled trials. We critically and comparatively analyzed the outcomes for each primary endpoint based on the intervention, reflected by the positive βhCG test, clinical pregnancy, vital pregnancy, ongoing pregnancy, live birth, live birth at 4 weeks, and multiple pregnancies.

**Results:**

Six randomized controlled trials were included in the quality assessment as priority manuscripts, revealing an 83.3% low risk of bias. Follitropin delta led to non-significant differences in each parameter of interest from positive βhCG test (691; 53.44% vs. 602; 46.55%), ongoing pregnancies (603; 53.79% vs. 518; 46.20%), clinical and vital pregnancies (1,073; 52.80% vs. 959; 47.19%), to live birth and at 4 weeks (595; 54.14% vs. 504; 45.85%) with only 2 losses, and even multiple pregnancies (8; 66.66% vs. 4; 33.33%). However, follitropin delta was well-tolerated among hypo- and hyper-responders without significant risk of ovarian hyperstimulation syndrome and/or preventive interventions in contrast with follitropin alfa or follitropin beta.

**Conclusion:**

The personalized individualized-based algorithm dosing with follitropin delta is non-inferior to conventional follitropin alfa or follitropin beta. It is as effective in promoting a similar response in women without significant comparable adverse effects.

**Supplementary Information:**

The online version contains supplementary material available at 10.1007/s10815-024-03146-1.

## Introduction

The pioneering work on gonadotropins [1, 2] corroborated with the joint applications in translational medicine [3] paved the way towards synthesizing novel recombinant human follicle-stimulating hormones (r-hFSHs) of high purity through different biological processes [4]. These preparations [5] advanced as essential parts for ovarian stimulation and response of in vitro fertilization (IVF)/intracytoplasmic sperm injection (ICSI) within current assisted reproductive technologies (ART) protocols [3].

Given the inter-individual heterogeneity and variability across ethnic populations [6–9], the need for predictive factors adapted for patient stratification in acquiring high-quality embryos for transfer was widely accepted. Thus, it arose the idea of shifting from standardization to individualization to alleviate the risks of cycle cancelation, poor ovarian stimulation, and ovarian hyperstimulation syndrome (OHSS) [10–12] and culminated in overcoming the initial limitations of implementation in clinical practice [10, 12–14].

Follitropin delta (Rekovelle®) produced by Ferring Pharmaceuticals is uniquely derived from a cell line of human fetal retinal origin (PER.C6; Crucell) [3, 15, 16] as the latest r-hFSH used for ovarian stimulation that differentiates from other r-FSHs by their Chinese hamster ovary (CHO) cell lines origin [3, 4, 16, 17]. This drug retains an identical amino acid sequence in α and β subunits of which post-translational modifications resemble the glycosylation profile of native human FSH [15, 16], integrating a high degree of tri- and tetra-sialylated glycans and α2,3- and α2,6-linked sialic acid [16]. Since it blocks the asialoglycoprotein receptor (ASGPR) in the liver [4], this biological process explains the lower clearance, similar half-life bioavailability [15, 16], and higher ovarian stimulation than other rFSH [15].

While conventional r-FSHs uses international reference standard (IU) calculation with Steelman–Pohley bioassay, follitropin delta is dosed by mass (µg) because of the specific bioactivity, with an established dosing algorithm following pharmaco-kinetics/dynamic modeling exercise [15]. In light with the European Society of Human Reproduction and Embryology (ESHRE) guidelines recommendations [14], the approved dosing algorithm comprises both the body weight and anti-Müllerian hormone (AMH) level that impacts the drug exposure distribution volume and ovarian response [18–21].

Although randomized controlled trials represent the best approach to compare interventional treatments, they are limited by generalizability and strict inclusion criteria of patients under specific conditions [22]. Considering this argument, our purpose in this primary systematic review is to shed light on the non-inferiority potential by comparing the pregnancy outcomes represented by the established primary endpoints (positive βhCG test, clinical pregnancy, vital pregnancy, ongoing pregnancy, live birth, live birth at 4 weeks, and multiple pregnancies) among women undergoing conventional ovarian stimulation with follitropin alfa/follitropin beta compared with the individualized dosing algorithm of follitropin delta.

## Materials and methods

### Methodology and registration

This protocol was designed to adhere to the Preferred Reporting Items for Systematic Reviews and Meta-Analyses (PRISMA) 2020 guidelines [23] and registered in the International Prospective Register of Systematic Reviews database (PROSPERO) (CRD42024512792).

### Ethical approval

This systematic review did not required Institutional Review Board (IRB) consent or evaluation from another panel of expertise, as research data were extracted from published studies.

### Database search

Inquiries in distinct bibliographic academic databases that include PubMed-MEDLINE - United States National Library of Medicine (NLM, 1996), Web of Science™ (WOS) (Clarivate Analytics, 1997), Cochrane Database of Systematic Reviews (CDSR) (Cochrane Library, 1993), and Scopus (Elsevier, 2004) [24] were performed to identify, rank, and analyze potential suitable studies using MeSH (Medical Subject Headings) terms. The searches were restricted between December 12, 2016, and January 21, 2024. The date of inception represents the interval from authorization by the European Medicines Agency (EMA) and the current state of knowledge. We used synonyms, from the marketing name „Rekovelle” to “Follitropin Delta” and “FE 999049” to even abbreviations of the two projects entitled “The Evidence-based Stimulation Trial with Human rFSH in Europe and Rest of World” (“ESTHER-1”) and “ESTHER-2.” “Follitropin Delta” and “FE 999049″ are listed as [Supplementary Concept] and not Major Topics [Majr] that were introduced on June 11, 2017, respectively, July 8, 2016, according to the NLM official website. Both can be identified with the following credentials: MeSH Unique ID and Registry Number: C000620228-076WHW89TW and C000608977.

### Strategy and strings

We used a dedicated terminology for Search #1 that relies on sole vocabulary components “Follitropin Delta” OR “FE 999049” OR “Rekovelle” followed by “ESTHER-1” OR “ESTHER-2” and complex clusters using Boolean operators (“AND” or “OR”) for Search #2. The complete string sets for each database are available in Supplementary File 1.

### Study selection

References retrieved were imported to Mendeley – Reference Management Software (v. 1.19.8) (Elsevier, 2013) and de-duplicated by accessing the “check for duplicates” function followed by a second manual screening for accuracy. O.-D.I. and T.A. assessed the titles ± abstracts of each record for relevance and tangency with the scope, while the list of articles considered was subsequently scanned by assessing the entire content. Divergent opinions were resolved by common consent between each author. A tabular form for the retrieved records can be consulted in Supplementary File 2.

### Objectives

The main resolution of this manuscript consists in answering the question of whether the third r-hFSH is superior to follitropin alfa/beta for one or multiple interest primary endpoints: positive βhCG test, clinical pregnancy, vital pregnancy, ongoing pregnancy, live birth, live birth at 4 weeks, and multiple pregnancies. We designed a Patient (P), Intervention (I), Comparator (C), and Outcome (O) (PICO) structure to develop the main research question and the criteria for inclusion and exclusion. The adopted PICO format is presented in Supplementary File 3.

### Data extraction

Series of evidence in a tabular format using Microsoft Excel 2010 (Microsoft Corporation, Redmond, WA, USA) for sorting and coding were independently extracted through a standardized form developed to characterize included studies by B.D., O.-D.I., and R.M. that describe methodological data: author’s first name, year of publication, journal, country or countries, participant’s age, study design and population, and outcome measures reported as number and percentage (%).

### Quality assessment

B.D. and O.-D.I. independently evaluated the quality of each included study using the Revised Cochrane risk-of-bias tool for randomized trials (RoB 2) [25] via https://sites.google.com/site/riskofbiastool/ (accessed on 28 January 2024) by providing guidance and different packages depending on the type of study. This tool provides a framework that classifies biases into five different domains: (1) bias arising from the randomization process; (2) bias due to deviations from intended interventions; (3) bias due to missing outcome data; (4) bias in measurement of the outcome; (5) bias in selection of the reported result. Answering to series of prompt questions varies from “yes,” “probably yes,” “probably no,” “no,” or “no information,” and calculating the overall risk may be categorized as “low risk of bias,” “some concerns,” and “high risk of bias.” For transparency of the RoB 2 evaluation, we employed the Risk-of-bias VISualization (ROBVIS) [26] in the present systematic review.

### Inclusion/exclusion criteria

Whether the manuscripts were structured respecting the IMRaD format, they had to be written in English, report original data, and be published in a peer-reviewed journal. Therefore, primary research from RCTs was considered if available as full-length articles.

## Results

### Studies reviewed

**Fig. 1:**
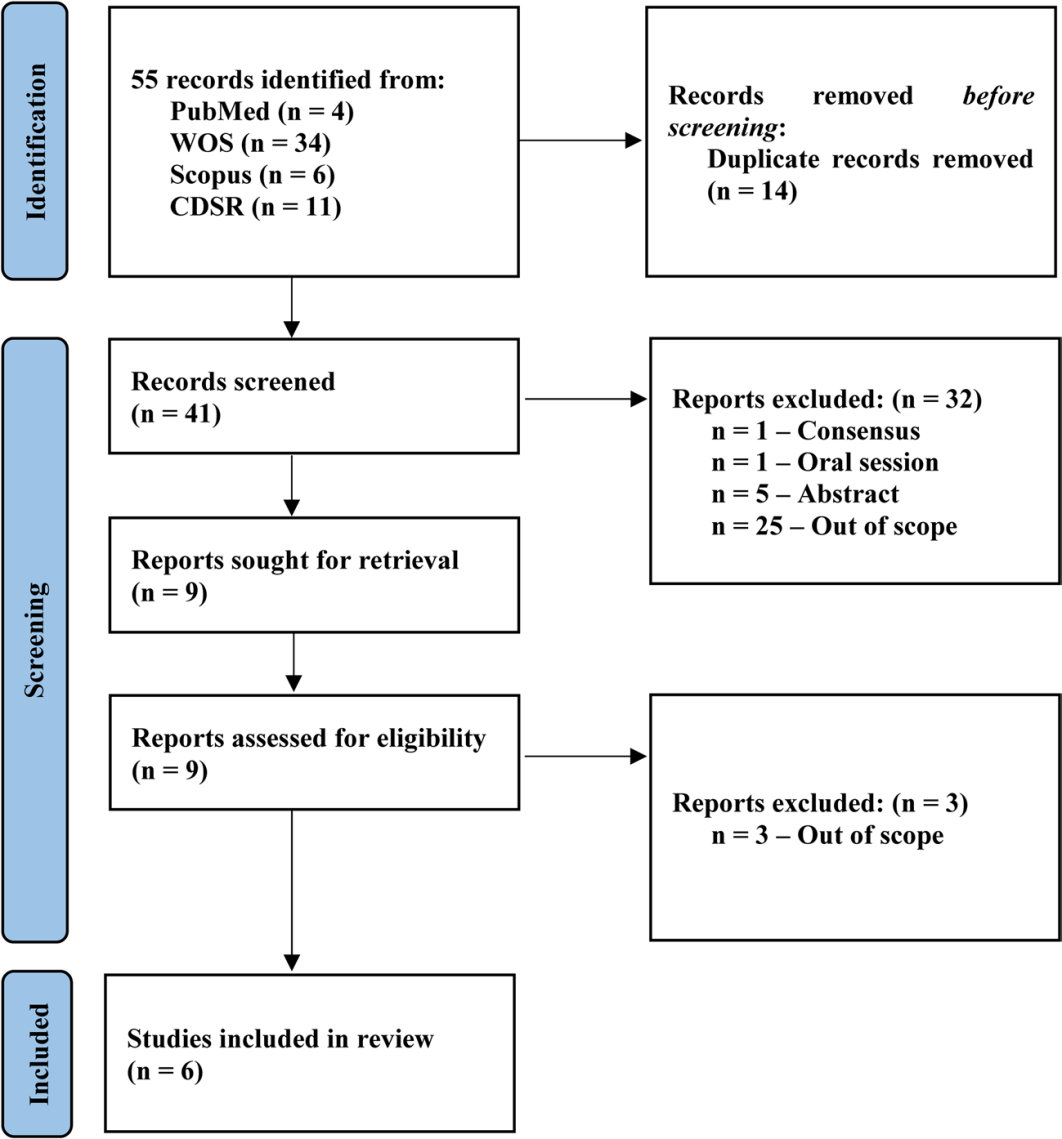
PRISMA flow diagram of the systematic review

From 55 papers identified between December 12, 2016, and January 21, 2024, including duplicates and studies that had no relevance to the primary research question, 14 were subsequently removed as being duplicates after review. Of the remaining 41 records subjected to full-text assessment, an additional 32 manuscripts were excluded during the first phase and other 3 during the second step, as detailed in the PRISMA flowchart in Fig. 1. The complete list with the individual and overall number of publications per year and lists of records can be consulted in the Supplementary File 4. Therefore, we retained six exclusive and multinational RCTs [27–32] conducted primarily in Asia (*n* = 4) and Europe (*n* = 2), *n* = 1 in 2017, *n* = 3 in 2021, and *n* = 2 in 2022.

All these RCTs have been previously registered in https://clinicaltrials.gov/ (accessed on January 30, 2024) with the following ID numbers: Andersen et al. [27], NCT01956110, 000004, 2013–001669-17 (EudraCT Number), U1111-1147–6826 (Other Identifier) (OTHER: WHO); Ishihara et al. [28], NCT02309671, 000124; Qiao et al. [29], NCT03296527, 000145; Ishihara et al. [30], NCT03228680, 000273; Sánchez et al. [31], NCT03564509, 2017–003810-13 (EudraCT Number), 000289; Yang et al. [32], NCT03296527, 000145. Excepting NCT03564509 and NCT02309671 which were in phase 2 of testing and enrolled *n* = 779 women, NCT03296527, NCT03296527, NCT03228680, and NCT01956110 were in phase 3 of development and recruited 3446, thus being randomized a total of 4225 women. RTCs’ overview with the associated results for the relevant parameters of interest may be consulted in Table 1 and Table 2.

**Table 1:** Methodological data of the studies included in this systematic review

First name of the author	Journal	Year of publication	Country	Study design	Study population	Age of participants, and mean
Andersen et al. [27]	*Fertility and Sterility*	2017	Belgium, Brazil, Canada, Czech Republic, Denmark, France, Italy, Poland, Russia, Spain, UK	Randomized, controlled, assessor-blinded, international, multicenter, non-inferiority trial	1326 665, F∆ 661, Fα	18–40 yo 33.4 ± 3.9 vs. 33.2 ± 3.9 < 35 yo 394 (59.2) vs. 392 (59.3) 35–37 yo 161 (24.2) vs. 167 (25.3) 38–40 yo 110 (16.5) vs. 102 (15.4)
Ishihara et al. [28]	*Fertility and Sterility*	2021	Japan	Randomized, controlled, assessor-blind, parallel groups, multicenter phase 2 trial	158 117, F∆ 6 µg/d, 37 9 µg/d, 40 12 µg/d, 40 41, Fβ 150 IU/d, 41	20–39 yo 6 µg/d—33.1 ± 3.8 9 µg/d—33.9 ± 3.4 12 µg/d—34.6 ± 3.4 150 IU/d—33.3 ± 3.9
Qiao et al. [29]	*Human Reproduction*	2021	Mainland China, South Korea, Taiwan, Vietnam	Randomized, controlled, assessor-blind, parallel groups, multicenter, non-inferiority trial	1009 499, F∆ 510, Fα	20–40 yo 31.1 ± 3.7 vs. 31.2 ± 3.8^a^ < 35 yo 394 (79.0) vs. 396 (77.6) 35–37 yo 85 (17.0) vs. 86 (16.9) 38–40 yo 20 (4.0) vs. 28 (5.5)
Ishihara et al. [30]	*Reproductive Biomedicine Online*	2021	Japan	Randomized, controlled, assessor-blind, multicenter, non-inferiority trial	347 170, F∆ 177, Fα	20–40 yo 34.2 ± 3.5 vs. 34.0 ± 3.4^a^ < 35 yo 88 (51.8) vs. 93 (52.5) 35–37 yo 51 (30.0) vs. 55 (31.1) 38–40 yo 31 (18.2) vs. 29 (16.4)
Sánchez et al. [31]	*Human Reproduction*	2022	Belgium, Czech Republic, Denmark, Spain, UK	Randomized, double-blind, multicenter, parallel-groups, phase 2, dose-range trial	619 515, F∆ 1 µg/d, 104 2 µg/d, 101 4 µg/d, 99 8 µg/d, 107 12 µg/d, 104 P, 104	30–42 yo 35.6 ± 3.3^a^
Yang et al. [32]	*Reproductive Biology and Endocrinology*	2022	Mainland China, South Korea, Taiwan, Vietnam	Randomized, controlled, assessor-blind, parallel groups, multicenter, phase 3, non-inferiority trial	759 378, F∆ 381, Fα	20–40 yo 30.6 ± 3.6 vs. 30.8 ± 3.6^a^ < 35 yo 312 (82.5) vs. 313 (82.2) 35–37 yo 55 (14.6) vs. 52 (13.6) 38–40 yo 11 (2.9) vs. 16 (4.2)

*yo*, years old; ^a^overall mean years; *P*, placebo

**Table 2:** Results of the established primary endpoints

First name of the author	Positive βhCG	Clinical pregnancy	Vital pregnancy	Ongoing pregnancy	Live birth	Live birth at 4 weeks	Multiple pregnancies
Andersen et al. [27]	Per started cycle 257 (38.6) vs 266 (40.2)	Per started cycle 232 (34.9) vs 241 (36.5)	Per started cycle 211 (31.7) vs 221 (33.4)	Per started cycle 204 (30.7) vs 209 (31.6)	Per started cycle 198 (29.8) vs 203 (30.7)	Per started cycle 198 (29.8) vs 201 (30.4)	per started cycle 4 (2.0) vs 8 (3.8)
Ishihara et al. [28]	Per started cycle 10/37 (27) vs 10/40 (25) vs 14/40 (35) vs 9/41 (22) Per cycle with transfer 10/26 (38) vs 10/31 (32) vs 14/32 (44) vs 9/31 (29)	Per started cycle 9/37 (24) vs 8/40 (20) vs 13/40 (33) vs 8/41 (20) Per cycle with transfer 9/26 (35) vs 8/31 (26) vs 13/32 (41) vs 8/31 (26)	Per started cycle 7/37 (19) vs 8/40 (20) vs 10/40 (25) vs 6/41 (15) Per cycle with transfer 7/26 (27) vs 8/31 (26) vs 10/32 (31) vs 6/31 (19)	Per started cycle 6/37 (16) vs 7/40 (18) vs 10/40 (25) vs 6/41 (15) Per cycle with transfer 6/26 (23) vs 7/31 (23) vs 10/32 (31) vs 6/31 (19)	Per started cycle 6/37 (16) vs 7/40 (18) vs 9/40 (23) vs 6/41 (15) Per cycle with transfer 6/26 (23) vs 7/31 (23) vs 9/32 (28) vs 6/31 (19)	Per started cycle 6/37 (16) vs 7/40 (18) vs 9/40 (23) vs 6/41 (15) Per cycle with transfer 6/26 (23) vs 7/31 (23) vs 9/32 (28) vs 6/31 (19)	NS
Qiao et al. [29]	208 (41.7) vs 180 (35.3)	180 (36.1) vs 159 (31.2)	NS	156 (31.3) vs 131 (25.7)	156 (31.3) vs 126 (24.7)	156 (31.3) vs 126 (24.7)	NS
Ishihara et al. [30]	NS	Per started cycle 43 (25.3) vs 42 (23.7) Per cycle with transfer 43 (31.9) vs 42 (29.8)	NS	Per started cycle 40 (23.5) vs 34 (19.2) Per cycle with transfer 40 (29.6) vs 34 (24.1)	Per started cycle 40 (23.5) vs 33 (18.6) Per cycle with transfer 40 (29.6) vs 33 (23.4)	Per started cycle 40 (23.5) vs 33 (18.6) Per cycle with transfer 40 (29.6) vs 33 (23.4)	NS
Sánchez et al. [31]	34.4 vs 39.1 vs 45.4 vs 47.9 vs 41.2 vs 49.8	NS	28.4 vs 30.1 vs 41.3 vs 40.3 vs 35.3 vs 42.9	28.4 vs 29.1 vs 39.2 vs 37.4 vs 30.4 vs 42.9	NS	NS	NS
Yang et al. [32]	158/378 (41.8) vs 138/381 (36.2)	134/378 (35.4) vs 120/381 (31.5)	120/378 (31.7) vs 106/381 (27.8)	117/378 (31.0) vs 98/381 (25.7)	117/378 (31.0) vs 97/381 (25.5)	117/378 (31.0) vs 97/381 (25.5)	NS

*NS* not specified

### Risk of bias assessment

**Fig. 2:**
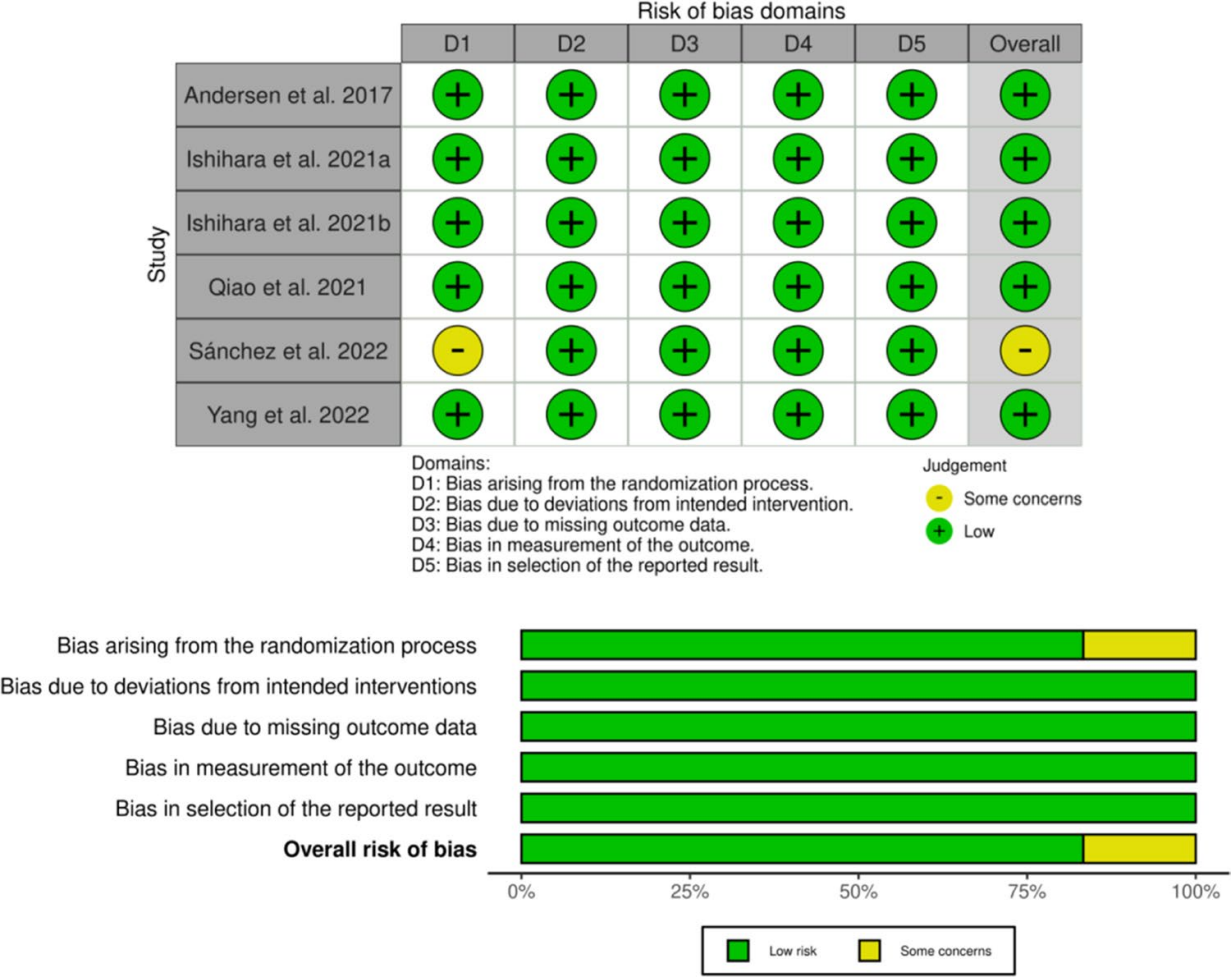
Overall assessment quality of the RTCs based on the ROB-2 tool

There has been an overall low risk (green color) of bias with an 83.3% quality on the assignment to the intention-to-treat (ITT), with only 16.7% indicating some concerns (yellow color) on one study [31] in D1 attributable to the “Bias arising from the randomization process” due to scarcity of baseline data to compare the differences between the groups and enrollment of underweight, overweight, and obese participants (Fig. 2).

### Positive βhCG

Positive serum βhCG at 13–15 days after blastocyst transfer was established in five [27–29, 31, 32] out of six [30] eligible RCTs and totals 1293 confirmed tests. Precisely, 691 (53.44%) women experienced OS with individualized follitropin delta in doses ranging from 1 µg to 12 µg/d guided by the AMH concentration. On the other hand, 602 (46.55%) resulted from the administration of the other two conventional r-FSHs, from which 584 (97%) of follitropin alfa and 18 (2.99%) of follitropin beta. However, the study of Sánchez et al. [31] which relied on adding choriogonadotropin beta (CG beta) and observed a negative response in contrast with the placebo (odds ratio - OR vs. placebo; 0.53; confidence interval - CI 95% (0.30; 0.93); *P* = 0.0264). Although Ishihara et al. [30] conducted a similar study with the same primary endpoints [28], they did not report the associated data. In their prior experience, they have recorded elevated levels of βhCG among 68 participants (34/34) allocated to receive 6 µg/d (10/10), 9 µg/d (10/10), and 12 µg/d (14/14) per started cycle and cycle with transfer than the arm with follitropin beta with 18 (9/9) women exhibiting positive serum βhCG on 150 IU/daily.

### Ongoing pregnancy

Referring to the ongoing pregnancy as having at least one intrauterine viable fetus 10–11 weeks after blastocyst transfer [27–32], it accounts for an overall 1121 ongoing pregnancies, as 603 (53.79%) were associated with follitropin delta. Moreover, 518 (46.20%) correlate with conventional dosing, 438 (84.55%), follitropin alfa, and 80 (15.44%), follitropin beta. The proportion for the latter was 40 (6/34) per started cycle and 40 (6/34) per cycle with transfer, whereas for the pre-defined fixed-dose were 6 µg/d (6/6), 9 µg/d (7/7), and 12 µg/d (10/10) [28, 30]. The maximum allowed dose of 12 µg/daily of follitropin delta promoted the highest response enabling 20 (10/10) ongoing pregnancies [28], but exerts a dose-treatment effect because of the OR vs. placebo of 0.58; CI 95% (0.33; 1.03) with a *P* = 0.0650) [31] as it decreases depending on the dose concentration.

### Vital and clinical pregnancy

In the context of vital and clinical pregnancies regarded as at least one intrauterine gestational sac with a fetal heartbeat 5–6 weeks after transfer, three RCTs [27, 28, 32] have investigated these parameters. The remaining either emphasize the rates of clinical [29, 30] or vital pregnancies [31]. It facilitated around 2032 pregnancies, particularly 1312 clinical and 720 vital pregnancies, irrespective of the intervention drug. Ovarian stimulation with follitropin delta led to obtaining 1073 (52.80%) combined cases, while convetional to 959 (47.19%), from which follitropin alfa to 847 (41.68%) and follitropin beta to 112 (5.51%). Analogous to the investigations conducted by Ishihara et al. [28, 30], they highlighted that 308 pregnancies correspond to the ratio of 154 (98/56)/154 (98/56) cases related to the per started cycles and cycle with transfer for follitropin delta and follitropin beta. The ovarian response relying on the distinctive doses for both parameters was as follows: 6 µg/d–16 (9/7), 9 µg/d–16 (8/8), 12 µg/d–23 (13/10), and 15 IU/d–14 (8/6). The findings of Sánchez et al. [31] reflected a non-significant difference following 4 µg as it has the highest effect compared with placebo (OR vs. placebo; 0.93; CI 95% (0.53; 1.64) with a *P* = 0.8116).

### Live birth and at 4 weeks

By defining the live birth as having at least one live neonate who subsequently has reached 4 weeks after birth, five teams [27–30, 32] calculated the rates, except for one study [31]. From a cumulative 1099 births, 595 (54.14%) were attributed to follitropin delta, and 504 (45.85%) to both conventional r-FHS, particularly 426 (38.76%) to follitropin alfa and 78 (7.09%) to follitropin beta. With an equivalent number of live neonates at 4 weeks, two women had losses without further details in this context [27]. As detailed earlier, the ratio per started cycle and cycle with transfer was identical, 6 µg/d (6/6), 9 µg/d (7/7), 12 µg/d (9/9) and 150 IU/d (6/6) according to the Ishihara et al. [28]. Dose adjustments of follitropin beta did not differ in terms of neonatal death in comparison with follitropin delta, 80 (40/40) and 66 (33/33) [30].

### Multiple pregnancies

Andersen et al. [27] are the sole investigators who monitored the likelihood of multiple pregnancies and observed that 12 were obtained following ovarian stimulation: 8 (66.66%) in the follitropin alfa and half in the follitropin delta (33.33%).

## Discussion

In this first systematic review, we aimed to summarize evidence on the non-inferiority potential of individualized algorithm-based follitropin delta administration in terms of pregnancy outcomes compared with conventional dosing. It has been estimated that a daily dose of 10.0 µg follitropin delta corresponds to 150 IU/d [33] by possessing well tolerability up to 12–24 µg in Chinese women [34] with respect to the relatively same percentages on pregnancy outcomes [35, 36].

Considering the low immunogenicity among women undergoing multiple stimulation cycles [35], the post hoc analyses emphasize a similar number of oocytes yielded and retrieved [33, 35, 37], irrespective of ovarian reserve, which interestingly was subsequently contradicted on further examinations [36]. Regardless of the reported mean retrieval of > 10 oocytes [38–40] except in one instance [41] and that > 40% achieved the established optimal range between 8 and 14 oocytes [38, 40, 41], the pregnancy outcomes did not reveal significant discrepancies between the groups, neither cumulative or per fresh ET [38–42].

Of note is that Asian women exhibit a higher risk of OHSS owing to the ethnic-related differences in weight than European females [36, 37, 43], but in a predictable dose-dependent and dose-exposure proportionality [44]. This observation refutes additional evidence on reduced risk of moderate/severe OHSS and preventive interventions in participants that followed ovarian stimulation with follitropin delta [43, 45]. OHSS is a common iatrogenic complication reported in clinical trials. Most cases ranged from mild to moderate OHSS [38, 39, 46] or isolated severe [38] and circumstances of any grade OHSS where the incidence was higher than in the ESTHER-1, but with fewer moderate or severe cases [47].

Cumulative data highlight the safety profile of follitropin delta irrespective of the ovarian reserve as was tested in categories of patients regarded as normal, low, and high responders [36, 37] indicative by the AMH value [43] even though it appears to differ in a dose–response manner depending on the AMH via a r-hFSH and endocrine parameters-follicular development interconnection [19]. However, its variability across stages of the menstrual cycle through multiple measurements suggests a limited impact considering the number of oocytes ± 1 when AMH < 15 pmol/L attained and dose adjustments of ± 1.5 μg when AMH ≥ 15 pmol/L [48]. It is worth noting that previous studies have found that commercial AMH assays may exhibit an intercycle variation exceeding 20% and reach 163% [49–51], which was associated with a lack of standardized AMH tests [52–54]. This has first limited result comparisons, but this concern was resolved since current assays started to display concordance for gonadotrophin prescribing [55].

The MARCS trial results regarding a higher mean number of oocytes retrieved and good quality blastocysts [47] are conflicting with the reports of another study in which the authors argue the presence of a lower proportion of day 3 good and intermediate blastocysts [56]. Moreover, PROFILE [39] and DELTA [38] trials, in parallel with that who preceded [42] or compared the results obtained in the ESTHER programs [40, 41, 46], have reached congruent conclusions on primary and secondary outcomes. Even if ET was fresh or frozen during a woman’s first stimulation cycle, the rate of major congenital disorders remained lower throughout the first 4 weeks after birth [42].

More recent studies from members of the ESTHER group [57] or conducted on specific populations from Europe [58] and North America [59, 60] that might imply the concomitant administration of long gonadotropin-releasing hormone (GnRH) agonist [61] added further proof to the applicability into the “real-world” and extrapolation in clinical practice. Follitropin delta combined with menotropin [61] led to changes in endocrine and reproductive profile [57], while alone had no effect [58], and without notable clinical outcomes [57, 61]. It appears that even a combined approach necessitates OHSS preventive measures [61], besides being constantly reported and varying from early to moderate, even late and graded from moderate to severe [57].

Fertility nurses rated the GONAL-f pen injector to be less prone to handling errors than other injectors [62] as there was an isolated situation in which the women wrongfully exposed themselves for short term to 72 µg follitropin delta for 3 consecutive days which interestingly had no major adverse events (AEs) [63].

### Strengths and limitations of the study

This is the first systematic review conducted on this topic, and the quality of the RCTs included indicates a low risk of bias. However, it is important to note that there is a relatively low number of studies overall, and all of these have been predominantly conducted by the same team members.

## Conclusions

Based on all the aspects covered in this systematic review, it can be concluded that follitropin delta provides a more consistent response than follitropin alfa or beta, thus being non-inferior. Follitropin delta proved to be a reliable r-hFSH dedicated to ovarian stimulation. This manuscript consolidates the actual spectrum of knowledge and may assist clinicians and researchers in future studies to translate this in clinical practice.

### Supplementary Information

Below is the link to the electronic supplementary material.

Supplementary file1 (DOCX 26.5 KB)

## Data Availability

The datasets used and analyzed during the current study are available from the corresponding author on reasonable request.

## References

[1] Lunenfeld B, Bilger W, Longobardi S, Alam V, D’Hooghe T, Sunkara SK. The Development of Gonadotropins for Clinical Use in the Treatment of Infertility [Internet]. Front Endocrinol. 2019;10:429. 10.3389/fendo.2019.00429. 10.3389/fendo.2019.00429 PMC6616070 31333582

[2] Lunenfeld B. Gonadotropin stimulation: past, present and future. Reprod Med Biol [Internet]. 2012;11:11–25. 10.1007/s12522-011-0097-2. 29699102 10.1007/s12522-011-0097-2 PMC5906949

[3] Niederberger C, Pellicer A, Cohen J, Gardner DK, Palermo GD, O’Neill CL, et al. Forty years of IVF. Fertil Steril [Internet]. 2018;110:185-324.e5. 10.1016/j.fertnstert.2018.06.005. 30053940 10.1016/j.fertnstert.2018.06.005

[4] Dias J, Ulloa-Aguirre A. New human follitropin preparations: how glycan structural differences may affect biochemical and biological function and clinical effect. Front Endocrinol (Lausanne). 2021;12:636038. 33815292 10.3389/fendo.2021.636038 PMC8018285

[5] De Leo V, Musacchio MC, Di Sabatino A, Tosti C, Morgante G, Petraglia F. Present and future of recombinant gonadotropins in reproductive medicine [Internet]. Curr Pharm Biotechnol. 2012;13(3):379–91. http://www.eurekaselect.com/node/76486/article. Accessed 1 Mar 2024. 10.2174/138920112799361918 21657999

[6] Sharara FI, McClamrock HD. Differences in in vitro fertilization (IVF) outcome between white and black women in an inner-city, university-based IVF program. Fertil Steril. 2000;73:1170–3. 10856477 10.1016/S0015-0282(00)00524-0

[7] Purcell K, Schembri M, Frazier LM, Rall MJ, Shen S, Croughan M, et al. Asian ethnicity is associated with reduced pregnancy outcomes after assisted reproductive technology. Fertil Steril. 2007;87:297–302. 17081529 10.1016/j.fertnstert.2006.06.031

[8] Huddleston HG, Rosen MP, Lamb JD, Modan A, Cedars MI, Fujimoto VY. Asian ethnicity in anonymous oocyte donors is associated with increased estradiol levels but comparable recipient pregnancy rates compared with Caucasians. Fertil Steril. 2010;94:2059–63. 20056204 10.1016/j.fertnstert.2009.11.019

[9] Tabbalat AM, Pereira N, Klauck D, Melhem C, Elias RT, Rosenwaks Z. Arabian Peninsula ethnicity is associated with lower ovarian reserve and ovarian response in women undergoing fresh ICSI cycles. J Assist Reprod Genet. 2018;35:331–7. 29063502 10.1007/s10815-017-1071-7 PMC5845040

[10] Fauser BCJM, Diedrich K, Devroey P. Predictors of ovarian response: progress towards individualized treatment in ovulation induction and ovarian stimulation. Hum Reprod Update. 2008;14:1–14. 18006561 10.1093/humupd/dmm034

[11] Nelson SM. Biomarkers of ovarian response: current and future applications. Fertil Steril [Internet]. 2013;99:963–9. 10.1016/j.fertnstert.2012.11.051. 23312225 10.1016/j.fertnstert.2012.11.051

[12] La Marca A, Sunkara SK. Individualization of controlled ovarian stimulation in IVF using ovarian reserve markers: from theory to practice. Hum Reprod Update [Internet]. 2014;20:124–40. 10.1093/humupd/dmt037. 24077980 10.1093/humupd/dmt037

[13] Broekmans FJ, Kwee J, Hendriks DJ, Mol BW, Lambalk CB. A systematic review of tests predicting ovarian reserve and IVF outcome. Hum Reprod Update. 2006;12:685–718. 16891297 10.1093/humupd/dml034

[14] ESHRE Guidelines. Ovarian stimulation for IVF/ICSI. In: Guideline of the European society of human reproduction and embryology (2019) [Internet]. 2019. https://www.google.com/search?q=.+Available+at%3A+https%3A%2F%2F+www.Eshre.Eu%2F-%2FMedia%2FSitecore-Files%2FGuidelines%2FCos%2FEshre-Cos-Guideline_Final%0209102019_.Pdf%3FLa%3DEn%26Hash%3D2316ea35f8afd21c2fb193c33f3bdc272334c901&oq=.+Available+at%3A+http. Accessed 4 Mar 2024.

[15] Koechling W, Plaksin D, Croston GE, Jeppesen J V, Macklon KT, Andersen CY. Comparative pharmacology of a new recombinant FSH expressed by a human cell line. Endocr Connect [Internet]. 2017;6:297–305. https://www.ncbi.nlm.nih.gov/pubmed/28450423. Accessed 1 Mar 2024. 10.1530/EC-17-0067 PMC5510450 28450423

[16] Olsson H, Sandström R, Grundemar L. Different pharmacokinetic and pharmacodynamic properties of recombinant follicle-stimulating hormone (rFSH) derived from a human cell line compared with rFSH from a non-human cell line. J Clin Pharmacol [Internet]. 2014;54:1299–307. 10.1002/jcph.328. 24800998 10.1002/jcph.328

[17] Howles CM. Genetic engineering of human FSH (Gonal-F®). Hum Reprod Update [Internet]. 1996;2:172–91. 10.1093/humupd/2.2.172. 9079412 10.1093/humupd/2.2.172

[18] Arce J-C, Klein BM, Erichsen L. Using AMH for determining a stratified gonadotropin dosing regimen for IVF/ICSI and optimizing outcomes. In: Seifer DB, Tal R, editors. Anti-Müllerian hormone: biology, role in ovarian function and clinical significance. 1st ed. Nova Science Publishers, Inc; 2016. pp. 83–102.

[19] Bosch E, Nyboe Andersen A, Barri P, García-Velasco JA, de Sutter P, Fernández-Sánchez M, et al. Follicular and endocrine dose responses according to anti-Müllerian hormone levels in IVF patients treated with a novel human recombinant FSH (FE 999049). Clin Endocrinol (Oxf) [Internet]. 2015;83:902–12. 10.1111/cen.12864. 26202150 10.1111/cen.12864

[20] Rose TH, Röshammar D, Erichsen L, Grundemar L, Ottesen JT. Characterisation of Population Pharmacokinetics and Endogenous Follicle-Stimulating Hormone (FSH) Levels After Multiple Dosing of a Recombinant Human FSH (FE 999049) in Healthy Women. Drugs R D [Internet]. 2016;16:165–72. 10.1007/s40268-016-0126-z. 27139012 10.1007/s40268-016-0126-z PMC4875921

[21] Bergandi L, Canosa S, Carosso AR, Paschero C, Gennarelli G, Silvagno F, et al. Human recombinant FSH and its biosimilars: clinical efficacy, safety, and cost-effectiveness in controlled ovarian stimulation for in vitro fertilization. Pharmaceuticals (Basel). 2020;13:136. 32605133 10.3390/ph13070136 PMC7407829

[22] Suvarna V. Phase IV of drug development. Perspect Clin Res. 2010;1:57–60. 21829783 10.4103/2229-3485.71852 PMC3148611

[23] Page MJ, McKenzie JE, Bossuyt PM, Boutron I, Hoffmann TC, Mulrow CD, et al. The PRISMA 2020 statement: an updated guideline for reporting systematic reviews. BMJ [Internet]. 2021;372:n71. http://www.bmj.com/content/372/bmj.n71.abstract. Accessed 4 Mar 2024. 10.1136/bmj.n71 PMC8005924 33782057

[24] Falagas ME, Pitsouni EI, Malietzis GA, Pappas G. Comparison of PubMed, Scopus, Web of Science, and Google Scholar: strengths and weaknesses. FASEB J Off Publ Fed Am Soc Exp Biol. 2008;22:338–42. 10.1096/fj.07-9492LSF 17884971

[25] Sterne JAC, Savović J, Page MJ, Elbers RG, Blencowe NS, Boutron I, et al. RoB 2: a revised tool for assessing risk of bias in randomised trials. BMJ [Internet]. 2019;366:l4898. http://www.bmj.com/content/366/bmj.l4898.abstract. Accessed 4 Mar 2024. 10.1136/bmj.l4898 31462531

[26] McGuinness LA, Higgins JPT. Risk-of-bias VISualization (robvis): An R package and Shiny web app for visualizing risk-of-bias assessments. Res Synth Methods [Internet]. 2020;12:55–61. 10.1002/jrsm.1411. 32336025 10.1002/jrsm.1411

[27] Nyboe Andersen A, Nelson SM, Fauser BCJM, García-Velasco JA, Klein BM, Arce J-C, et al. Individualized versus conventional ovarian stimulation for in vitro fertilization: a multicenter, randomized, controlled, assessor-blinded, phase 3 noninferiority trial. Fertil Steril [Internet]. 2017;107:387-396.e4. 10.1016/j.fertnstert.2016.10.033. 27912901 10.1016/j.fertnstert.2016.10.033

[28] Ishihara O, Klein BM, Arce J-C, Kuramoto T, Yokota Y, Mukaida T, et al. Randomized, assessor-blind, antimüllerian hormone-stratified, dose-response trial in Japanese in vitro fertilization/intracytoplasmic sperm injection patients undergoing controlled ovarian stimulation with follitropin delta. Fertil Steril [Internet]. 2021;115:1478–86. 10.1016/j.fertnstert.2020.10.059. 33272623 10.1016/j.fertnstert.2020.10.059

[29] Qiao J, Zhang Y, Liang X, Ho T, Huang H-Y, Kim S-H, et al. A randomised controlled trial to clinically validate follitropin delta in its individualised dosing regimen for ovarian stimulation in Asian IVF/ICSI patients. Hum Reprod [Internet]. 2021;36:2452–62. 10.1093/humrep/deab155. 34179971 10.1093/humrep/deab155 PMC8373472

[30] Ishihara O, Arce J-C. Individualized follitropin delta dosing reduces OHSS risk in Japanese IVF/ICSI patients: a randomized controlled trial. Reprod Biomed Online [Internet]. 2021;42:909–18. 10.1016/j.rbmo.2021.01.023. 33722477 10.1016/j.rbmo.2021.01.023

[31] Fernández Sánchez M, Višnová H, Larsson P, Yding Andersen C, Filicori M, Blockeel C, et al. A randomized, controlled, first-in-patient trial of choriogonadotropin beta added to follitropin delta in women undergoing ovarian stimulation in a long GnRH agonist protocol. Hum Reprod [Internet]. 2022;37:1161–74. 10.1093/humrep/deac061. 35451013 10.1093/humrep/deac061 PMC9156848

[32] Yang R, Zhang Y, Liang X, Song X, Wei Z, Liu J, et al. Comparative clinical outcome following individualized follitropin delta dosing in Chinese women undergoing ovarian stimulation for in vitro fertilization /intracytoplasmic sperm injection. Reprod Biol Endocrinol. 2022;20:147. 36195924 10.1186/s12958-022-01016-y PMC9531501

[33] Arce J-C, Larsson P, García-Velasco JA. Establishing the follitropin delta dose that provides a comparable ovarian response to 150 IU/day follitropin alfa. Reprod Biomed Online [Internet]. 2020;41:616–22. https://www.sciencedirect.com/science/article/pii/S1472648320303771. Accessed 1 Mar 2024. 10.1016/j.rbmo.2020.07.006 32819842

[34] Shao F, Jiang Y, Ding S, Larsson P, Pinton P, Jonker DM. Pharmacokinetics and Safety of Follitropin Delta in Gonadotropin Down-Regulated Healthy Chinese Women. Clin Drug Investig. 2023;43:37–44. 36478528 10.1007/s40261-022-01232-9 PMC9834375

[35] Bosch E, Havelock J, Martin FS, Rasmussen BB, Klein BM, Mannaerts B, et al. Follitropin delta in repeated ovarian stimulation for IVF: a controlled, assessor-blind Phase 3 safety trial. Reprod Biomed Online [Internet]. 2019;38:195–205. 10.1016/j.rbmo.2018.10.012. 30594482 10.1016/j.rbmo.2018.10.012

[36] Višnová H, Papaleo E, Martin FS, Koziol K, Klein BM, Mannaerts B. Clinical outcomes of potential high responders after individualized FSH dosing based on anti-Müllerian hormone and body weight. Reprod Biomed Online [Internet]. 2021;43:1019–26. 10.1016/j.rbmo.2021.08.024. 34756645 10.1016/j.rbmo.2021.08.024

[37] Ishihara O, Nelson SM, Arce J-C. Comparison of ovarian response to follitropin delta in Japanese and White IVF/ICSI patients. Reprod Biomed Online [Internet]. 2022;44:177–84. 10.1016/j.rbmo.2021.09.014. 34799275 10.1016/j.rbmo.2021.09.014

[38] Porcu-Buisson G, Maignien C, Swierkowski-Blanchard N, Rongières C, Ranisavljevic N, Oger P, et al. Prospective multicenter observational real-world study to assess the use, efficacy and safety profile of follitropin delta during IVF/ICSI procedures (DELTA Study). Eur J Obstet Gynecol Reprod Biol. 2024;293:21–6. 38100937 10.1016/j.ejogrb.2023.12.011

[39] Blockeel C, Griesinger G, Rago R, Larsson P, Sonderegger YLY, Rivière S, et al. Prospective multicenter non-interventional real-world study to assess the patterns of use, effectiveness and safety of follitropin delta in routine clinical practice (the PROFILE study). Front Endocrinol (Lausanne). 2022;13:992677. 36619578 10.3389/fendo.2022.992677 PMC9815701

[40] Bachmann A, Kissler S, Laubert I, Mehrle P, Mempel A, Reissmann C, et al. An eight centre, retrospective, clinical practice data analysis of algorithm-based treatment with follitropin delta. Reprod Biomed Online [Internet]. 2022;44:853–7. 10.1016/j.rbmo.2021.12.013. 35193799 10.1016/j.rbmo.2021.12.013

[41] Kovacs P, Jayakumaran J, Lu Y, Lindheim SR. Comparing pregnancy rates following ovarian stimulation with follitropin-Δ to follitropin -α in routine IVF: A retrospective analysis. Eur J Obstet Gynecol Reprod Biol [Internet]. 2023;280:22–7. 10.1016/j.ejogrb.2022.11.006. 36375361 10.1016/j.ejogrb.2022.11.006

[42] Havelock J, Aaris Henningsen A-K, Mannaerts B, Arce J-C, Groups E-1 and E-2 T. Pregnancy and neonatal outcomes in fresh and frozen cycles using blastocysts derived from ovarian stimulation with follitropin delta. J Assist Reprod Genet [Internet]. 2021;38:2651–61. 10.1007/s10815-021-02271-5 10.1007/s10815-021-02271-5 PMC8581102 34254211

[43] Fernández-Sánchez M, Visnova H, Yuzpe A, Klein BM, Mannaerts B, Arce J-C. Individualization of the starting dose of follitropin delta reduces the overall OHSS risk and/or the need for additional preventive interventions: cumulative data over three stimulation cycles. Reprod Biomed Online [Internet]. 2019;38:528–37. 10.1016/j.rbmo.2018.12.032. 30713022 10.1016/j.rbmo.2018.12.032

[44] Olsson H, Sandström R, Bagger Y. Dose-exposure proportionality of a novel recombinant follicle-stimulating hormone (rFSH), FE 999049, derived from a human cell line, with comparison between Caucasian and Japanese women after subcutaneous administration. Clin Drug Investig [Internet]. 2015;35:247–53. 10.1007/s40261-015-0276-8. 25773354 10.1007/s40261-015-0276-8 PMC4368841

[45] Višnová H, Papaleo E, Martin FS, Koziol K, Klein BM, Mannaerts B. Clinical outcomes of potential high responders after individualized FSH dosing based on anti-Müllerian hormone and body weight. Reprod Biomed Online. 2021;43:1019–26. 34756645 10.1016/j.rbmo.2021.08.024

[46] Doroftei B, Ilie O-D, Dabuleanu A-M, Diaconu R, Maftei R, Simionescu G, et al. Follitropin delta as a state-of-the-art incorporated companion for assisted reproductive procedures: A two year observational study. Medicina. 2021;57:379. 33919919 10.3390/medicina57040379 PMC8070935

[47] Bissonnette F, MinanoMasip J, Kadoch I-J, Librach C, Sampalis J, Yuzpe A. Individualized ovarian stimulation for in vitro fertilization: a multicenter, open label, exploratory study with a mixed protocol of follitropin delta and highly purified human menopausal gonadotropin. Fertil Steril [Internet]. 2021;115:991–1000. 10.1016/j.fertnstert.2020.09.158. 33267959 10.1016/j.fertnstert.2020.09.158

[48] Nelson SM, Larsson P, Mannaerts BMJL, Nyboe Andersen A, Fauser BCJM. Anti-Müllerian hormone variability and its implications for the number of oocytes retrieved following individualized dosing with follitropin delta. Clin Endocrinol (Oxf) [Internet]. 2019;90:719–26. 10.1111/cen.13956. 30801744 10.1111/cen.13956

[49] Bungum L, Tagevi J, Jokubkiene L, Bungum M, Giwercman A, Macklon N, et al. The impact of the biological variability or assay performance on amh measurements: A prospective cohort study with AMH tested on three analytical assay-platforms. Front Endocrinol (Lausanne) [Internet]. 2018;9:603. https://pubmed.ncbi.nlm.nih.gov/30459709. Accessed 1 Mar 2024. 10.3389/fendo.2018.00603 PMC6232665 30459709

[50] Gorkem U, Togrul C. Is There a Need to Alter the Timing of Anti-Müllerian Hormone Measurement During the Menstrual Cycle? Geburtshilfe Frauenheilkd. 2019;79:731–7. 31303661 10.1055/a-0840-3817 PMC6620182

[51] Melado L, Lawrenz B, Sibal J, Abu E, Coughlan C, Navarro AT, et al. Anti-müllerian Hormone During Natural Cycle Presents Significant Intra and Intercycle Variations When Measured With Fully Automated Assay. Front Endocrinol (Lausanne). 2018;9:686. 30542322 10.3389/fendo.2018.00686 PMC6278633

[52] Iliodromiti S, Salje B, Dewailly D, Fairburn C, Fanchin R, Fleming R, et al. Non-equivalence of anti-Müllerian hormone automated assays-clinical implications for use as a companion diagnostic for individualised gonadotrophin dosing. Hum Reprod [Internet]. 2017;32:1710–5. https://pubmed.ncbi.nlm.nih.gov/28854583. Accessed 1 Mar 2024. 10.1093/humrep/dex219 PMC5850658 28854583

[53] Magnusson Å, Oleröd G, Thurin-Kjellberg A, Bergh C. The correlation between AMH assays differs depending on actual AMH levels. Hum Reprod Open. 2017;2017:hox026. 30895238 10.1093/hropen/hox026 PMC6277007

[54] ACOG Committee Opinion No. 773 Summary: The Use of Antimüllerian Hormone in Women Not Seeking Fertility Care. Obstet Gynecol. 2019;133:840–1. 30913192 10.1097/AOG.0000000000003163

[55] La Marca A, Tolani AD, Capuzzo M. The interchangeability of two assays for the measurement of anti-Müllerian hormone when personalizing the dose of FSH in in-vitro fertilization cycles. Gynecol Endocrinol Off J Int Soc Gynecol Endocrinol. 2021;37:372–6. 10.1080/09513590.2020.1810659 32856971

[56] Haakman O, Liang T, Murray K, Vilos A, Vilos G, Bates C, et al. In vitro fertilization cycles stimulated with follitropin delta result in similar embryo development and quality when compared with cycles stimulated with follitropin alfa or follitropin beta. F&S Reports [Internet]. 2021;2:30–5. 10.1016/j.xfre.2020.12.002. 34223270 10.1016/j.xfre.2020.12.002 PMC8244387

[57] Sánchez MF, Larsson P, Serrano MF, Bosch E, Velasco JAG, López ES, et al. Live birth rates following individualized dosing algorithm of follitropin delta in a long GnRH agonist protocol. Reprod Biol Endocrinol. 2023;21:45. 37194068 10.1186/s12958-023-01090-w PMC10185461

[58] Gazzo I, Bovis F, Colia D, Sozzi F, Costa M, Anserini P, et al. Algorithm vs clinical experience: controlled ovarian stimulations with follitropin delta and individualised doses of follitropin alpha/beta. Reprod Fertil [Internet]. 2024;5:e230045. https://raf.bioscientifica.com/view/journals/raf/5/1/RAF-23-0045.xml. Accessed 29 Feb 2024. 10.1530/RAF-23-0045 PMC10959055 38330591

[59] Arab S, Frank R, Ruiter J, Dahan MH. How to dose follitropin delta for the first insemination cycle according to the ESHRE and ASRM guidelines; a retrospective cohort study. J Ovarian Res [Internet]. 2023;16:24. 10.1186/s13048-022-01079-w. 36707880 10.1186/s13048-022-01079-w PMC9883945

[60] Yacoub S, Cadesky K, Casper RF. Low risk of OHSS with follitropin delta use in women with different polycystic ovary syndrome phenotypes: a retrospective case series. J Ovarian Res [Internet]. 2021;14:31. 10.1186/s13048-021-00773-5. 33579321 10.1186/s13048-021-00773-5 PMC7881448

[61] Duarte-Filho OB, Miyadahira EH, Matsumoto L, Yamakami LYS, Tomioka RB, Podgaec S. Follitropin delta combined with menotropin in patients at risk for poor ovarian response during in vitro fertilization cycles: a prospective controlled clinical study. Reprod Biol Endocrinol. 2024;22:7. 38166856 10.1186/s12958-023-01172-9 PMC10759374

[62] Longobardi S, Seidler A, Martins J, Beckers F, MacGillivray W, D’Hooghe T. An evaluation of the use and handling errors of currently available recombinant human follicle-stimulating hormone pen injectors by women with infertility and fertility nurses. Expert Opin Drug Deliv [Internet]. 2019;16:1003–14. 10.1080/17425247.2019.1651290. 31411099 10.1080/17425247.2019.1651290

[63] Baldini GM, Mastrorocco A, Sciorio R, Palini S, Dellino M, Cascardi E, et al. Inadvertent administration of 72 µg of Follitropin-Δ for three consecutive days does not appear to be dangerous for poor responders: a case series. J Clin Med. 2023;12:5202. 10.3390/jcm12165202 PMC10456029 37629245

