# Supplementary material for: The pregnancy outcomes among women receiving individualized algorithm dosing with follitropin delta: a systematic review of randomized controlled trials
Source: J Assist Reprod Genet. 2024 May 29;41(7):1851–61. doi: 10.1007/s10815-024-03146-1 (PMC11263530; doi:10.1007/s10815-024-03146-1)
Supplement: Supplementary file 1 — Supplementary file1 (DOCX 26.5 KB) [file 10815_2024_3146_MOESM1_ESM.docx]

**Appendix**

**Supplementary File 1.** Extensive searching model

Main search keywords:

Follitropin Delta, FE 999049, Rekovelle

**1. PubMed-MEDLINE – United States (NLM, 1996)**

*1.1. Detailed searching strings:*

((((Follitropin Delta) OR (FE 999049)) OR (Rekovelle)) AND (ESTHER-1)) OR (ESTHER-2) Filters: Randomized Controlled Trial, Humans, English, from 2016/12/12 - 2024/1/21

((("follitropin delta"[Supplementary Concept] OR "follitropin delta"[All Fields] OR ("fe 999049"[Supplementary Concept] OR "fe 999049"[All Fields] OR "fe 999049"[All Fields]) OR "Rekovelle"[All Fields]) AND "ESTHER-1"[All Fields]) OR "ESTHER-2"[All Fields]) AND ((randomizedcontrolledtrial[Filter]) AND (humans[Filter]) AND (2016/12/12:2024/1/21[pdat]) AND (english[Filter]))

*1.2. Filters applied for fields:*

ARTICLE LANGUAGE – English

SPECIES – Humans

PUBLICATION DATE – 12 December, 2016 – 21 January, 2024

ARTICLE TYPE – Randomized Controlled Trial

**2. Web of Science^TM^ (WOS) (Clarivate Analytics, 1997)**

*2.1. Detailed searching strings:*

Follitropin Delta (Topic) OR FE 999049 (Topic) OR Rekovelle (Topic) AND ESTHER-1 (Topic) OR ESTHER-2 (Topic) AND Randomized Controlled Trial (All Fields) and 2016 or 2017 or 2018 or 2019 or 2020 or 2021 or 2022 or 2023 or 2024 (Publication Years) and Article (Document Types) and Science Citation Index Expanded (SCI-EXPANDED) (Web of Science Index) and English (Languages)

*2.2. Filters applied for fields:*

LANGUAGE – ENGLISH

PUBLICATION YEARS – 2016 – 2024

DOCUMENT TYPES – ARTICLE

WEB OF SCIENCE CORE COLLECTION (INDEX) – SCIENCE CITATION INDEX EXPANDED (SCIE)

**3. Scopus (Elsevier, 2004)**

*3.1. Detailed searching strings:*

( TITLE-ABS-KEY ( follitropin AND delta ) ) OR ( TITLE-ABS-KEY ( fe 999049 ) ) OR ( TITLE-ABS-KEY ( rekovelle ) ) AND ( TITLE-ABS-KEY ( esther-1 ) ) OR ( TITLE-ABS-KEY ( esther-2 ) ) AND ( LIMIT-TO ( PUBYEAR , 2017 ) OR LIMIT-TO ( PUBYEAR , 2019 ) OR LIMIT-TO ( PUBYEAR , 2020 ) OR LIMIT-TO ( PUBYEAR , 2021 ) OR LIMIT-TO ( PUBYEAR , 2022 ) OR LIMIT-TO ( PUBYEAR , 2023 ) ) AND ( LIMIT-TO ( DOCTYPE , "ar" ) ) AND ( LIMIT-TO ( PUBSTAGE , "final" ) ) AND ( LIMIT-TO ( SRCTYPE , "j" ) ) AND ( LIMIT-TO ( LANGUAGE , "English" ) )

*3.2. Filters applied for fields:*

LANGUAGE – ENGLISH

YEAR – 2016 – 2024

SOURCE TYPE – JOURNAL

PUBLICATION STAGE – FINAL

DOCUMENT TYPE – ARTICLE

**4. Cochrane Database of Systematic Reviews (CDSR) (Cochrane Library, 1993)**

*4.1. Detailed searching strings:*

Follitropin Delta OR FE 999049 OR Rekovelle in Title Abstract Keyword AND ESTHER-1 OR ESTHER-2 in Title Abstract Keyword AND Randomized Controlled Trial in Title Abstract Keyword

*4.2. Filters applied for fields:*

YEAR – 2016 – 2024

LANGUAGE – English

**Supplementary File 2.** Cumulative list of returned entries depending on publication year

| **Publications per year** | **PubMed** | **WOS** | **Scopus** | **CDSR** |
| --- | --- | --- | --- | --- |
| 2016 | - | 1 | - | 3 |
| 2017 | 1 | 3 | 1 | 3 |
| 2018 | - | 1 | - | 1 |
| 2019 | 2 | 4 | 1 | 2 |
| 2020 | - | 3 | 1 | 2 |
| 2021 | 1 | 9 | 2 | - |
| 2022 | - | 8 | 1 | - |
| 2023 | - | 4 | - | - |
| 2024 | - | 1 | - | - |
| **Cumulative** | 4 | 34 | 6 | 11 |
| **Total** | 55 | | | |

**Supplementary File 3.** PICO table

| **Parameter** | **Inclusion criteria** | **Exclusion criteria** | **Data extraction** |
| --- | --- | --- | --- |
| **Patient, Population or Problem** | Adult females (>18-42 yo) diagnosed with:  - unexplained infertility  - tubal infertility  - EMS stage I/II  - partner-related factor infertility | - EMS stage III/IV  - history of recurrent miscarriage  - use of hormonal preparations during the last menstrual cycle before randomization | - general information  - participant’s age  - study design  - study population  - pregnancy outcomes |
| **Intervention or Exposure** | Ovarian stimulation in women undergoing IVF/ICSI with follitropin delta | Ovarian stimulation with follitropin alfa/follitropin beta with or without control/placebo group | - |
| **Comparison or Control** | Females adults (>18-42 yo) receiving individualized fixed-dose of follitropin delta in contrast with conventional follitropin alfa/beta or control/placebo group | A single group of analysis with drug intervention with or without control/placebo group | - |
| **Outcome** | Clinical results | Endocrine, embryology, and reproductive profiles | - positive βhCG test  - clinical pregnancy  - vital pregnancy  - ongoing pregnancy  - live birth  - multiple pregnancies |

yo – years old; EMS – endometriosis; IVF – in vitro fertilization; ICSI - intracytoplasmic sperm injection; βhCG – β-human chorionic gonadotropin

**Supplementary File 4.** Eligibility assessment processes

**First list of manuscripts examined**

| First author and year of publication | Article title | Eligible or not eligible? | Reason |
| --- | --- | --- | --- |
| Feferkorn et al. 2023 | The HERA (Hyper-response Risk Assessment) Delphi consensus definition of hyper-responders for in-vitro fertilization | Not eligible | Guideline |
| Fernández-Sánchez et al. 2018 | INDIVIDUALIZATION OF THE STARTING DOSE OF GONADOTROPIN REDUCES THE OVERALL OHSS RISK AND THE NEED OF PREVENTIVE INTERVENTIONS: CUMULATIVE DATA OVER THREE STIMULATION CYCLES | Not eligible | Oral session |
| Holtmann et al. 2019 | Follitropin delta for controlled ovarian stimulation: a one-year analysis | Not eligible | Abstract |
| Buur Rasmussen et al. 2016 | Low immunogenicity potential of follitropin delta, a recombinant FSH preparation produced from a human cell line: Results from phase 3 trials (ESTHER-1 and ESTHER-2) | Not eligible | Abstract |
| Nyboe Andersen et al. 2016 | Efficacy and safety of follitropin delta in an individualised dosing regimen: a randomised, assessor-blind, controlled phase 3 trial in IVF/ ICSI patients (ESTHER-1) | Not eligible | Abstract |
| Arce et al. 2020 | ESTABLISHING THE FOLLITROPIN DELTA DOSE PROVIDING COMPARABLE OVARIAN RESPONSE AS 150 IU/DAY FOLLITROPIN ALFA FOR CONTROLLED OVARIAN STIMULATION | Not eligible | Abstract |
| Ishihara et al. 2020 | SIMILAR OVARIAN RESPONSE WITH INDIVIDUALIZED FOLLITROPIN DELTA DOSING REGIMEN IN JAPANESE AND NON-JAPANESE IVF/ICSI PATIENTS | Not eligible | Abstract |
| Agresta et al. 2020 | Is it possible to apply trial outcomes to a real-world population? A novel approach to External Validity Analysis | Not eligible | Out of scope |
| Toftager et al. 2016 | Risk of severe ovarian hyperstimulation syndrome in GnRH antagonist versus GnRH agonist protocol: RCT including 1050 first IVF/ICSI cycles | Not eligible | Out of scope |
| Leijdekkers et al. 2019 | Do female age and body weight modify the effect of individualized FSH dosing in IVF/ICSI treatment? A secondary analysis of the OPTIMIST trial | Not eligible | Out of scope |
| Bungum et al. 2018 | The Impact of the Biological Variability or Assay Performance on AMH Measurements: A Prospective Cohort Study With AMH Tested on Three Analytical Assay-Platforms | Not eligible | Out of scope |
| Ebid et al. 2021 | Population PK-PD-PD Modeling of Recombinant Follicle Stimulating Hormone in In Vitro Fertilization/Intracytoplasmic Sperm Injection: Implications on Dosing and Timing of Gonadotrophin Therapy | Not eligible | Out of scope |
| Prabhudesai et al. 2021 | Central residues of FSHβ (89-97) peptide are not critical for FSHR binding: Implications for peptidomimetic design | Not eligible | Out of scope |
| Nelson et al. 2019 | Anti-Müllerian hormone variability and its implications for the number of oocytes retrieved following individualized dosing with follitropin delta | Not eligible | Out of scope |
| Doroftei et al. 2021 | Follitropin Delta as a State-of-the-Art Incorporated Companion for Assisted Reproductive Procedures: A Two Year Observational Study | Not eligible | Out of scope |
| La Marca et al. 2021 | The interchangeability of two assays for the measurement of anti-Müllerian hormone when personalizing the dose of FSH in in-vitro fertilization cycles | Not eligible | Out of scope |
| Greco et al. 2022 | COVID-19 - compliant - IVF: reorganized clinical practice taking into account the pandemic | Not eligible | Out of scope |
| Duarte-Filho et al. 2024 | Follitropin delta combined with menotropin in patients at risk for poor ovarian response during in vitro fertilization cycles: a prospective controlled clinical study | Not eligible | Out of scope |
| Iliodromiti et al. 2017 | Non-equivalence of anti-Müllerian hormone automated assays-clinical implications for use as a companion diagnostic for individualised gonadotrophin dosing | Not eligible | Out of scope |
| Bachmann et al. 2022 | An eight centre, retrospective, clinical practice data analysis of algorithm-based treatment with follitropin delta | Not eligible | Out of scope |
| Arab et al. 2023 | How to dose follitropin delta for the first insemination cycle according to the ESHRE and ASRM guidelines; a retrospective cohort study | Not eligible | Out of scope |
| Yacoub et al. 2021 | Low risk of OHSS with follitropin delta use in women with different polycystic ovary syndrome phenotypes: a retrospective case series | Not eligible | Out of scope |
| Kovacs et al. 2023 | Comparing pregnancy rates following ovarian stimulation with follitropin-Δ to follitropin-α in routine IVF: A retrospective analysis | Not eligible | Out of scope |
| Bissonnette et al. 2021 | Individualized ovarian stimulation for in vitro fertilization: a multicenter, open label, exploratory study with a mixed protocol of follitropin delta and highly purified human menopausal gonadotropin | Not eligible | Out of scope |
| Blockeel et al. 2022 | Prospective multicenter non-interventional real-world study to assess the patterns of use, effectiveness and safety of follitropin delta in routine clinical practice (the PROFILE study) | Not eligible | Out of scope |
| Shao et al. 2023 | Pharmacokinetics and Safety of Follitropin Delta in Gonadotropin Down-Regulated Healthy Chinese Women | Not eligible | Out of scope |
| Baldini et al. 2023 | Inadvertent Administration of 72 µg of Follitropin-Δ for Three Consecutive Days Does Not Appear to Be Dangerous for Poor Responders: A Case Series | Not eligible | Out of scope |
| Arce et al. 2020 | Establishing the follitropin delta dose that provides a comparable ovarian response to 150 IU/day follitropin alfa | Not eligible | Out of scope |
| Koechling et al. 2017 | Comparative pharmacology of a new recombinant FSH expressed by a human cell line | Not eligible | Out of scope |
| Havelock et al. 2021 | Pregnancy and neonatal outcomes in fresh and frozen cycles using blastocysts derived from ovarian stimulation with follitropin delta | Not eligible | Out of scope |
| Fernández-Sánchez et al. 2019 | Individualization of the starting dose of follitropin delta reduces the overall OHSS risk and/or the need for additional preventive interventions: cumulative data over three stimulation cycles | Not eligible | Out of scope |
| Bosch et al. 2019 | Follitropin delta in repeated ovarian stimulation for IVF: a controlled, assessor-blind Phase 3 safety trial | Not eligible | Out of scope |
| Fernández-Sánchez et al. 2023 | Live birth rates following individualized dosing algorithm of follitropin delta in a long GnRH agonist protocol | Eligible | - |
| Višnová et al. 2021 | Clinical outcomes of potential high responders after individualized FSH dosing based on anti-Müllerian hormone and body weight | Eligible | - |
| Ishihara et al. 2022 | Comparison of ovarian response to follitropin delta in Japanese and White IVF/ICSI patients | Eligible | - |
| Nyboe Andersen et al. 2017 | Individualized versus conventional ovarian stimulation for in vitro fertilization: a multicenter, randomized, controlled, assessor-blinded, phase 3 noninferiority trial | Eligible | - |
| Yang et al. 2022 | Comparative clinical outcome following individualized follitropin delta dosing in Chinese women undergoing ovarian stimulation for in vitro fertilization /intracytoplasmic sperm injection | Eligible | - |
| Qiao et al. 2021 | A randomised controlled trial to clinically validate follitropin delta in its individualised dosing regimen for ovarian stimulation in Asian IVF/ICSI patients | Eligible | - |
| Ishihara et al. 2021 | Randomized, assessor-blind, antimüllerian hormone-stratified, dose-response trial in Japanese in vitro fertilization/intracytoplasmic sperm injection patients undergoing controlled ovarian stimulation with follitropin delta | Eligible | - |
| Ishihara et al. 2021 | Individualized follitropin delta dosing reduces OHSS risk in Japanese IVF/ICSI patients: a randomized controlled trial | Eligible | - |
| Fernández-Sánchez et al. 2022 | A randomized, controlled, first-in-patient trial of choriogonadotropin beta added to follitropin delta in women undergoing ovarian stimulation in a long GnRH agonist protocol | Eligible | - |

**Second list of manuscripts examined**

| First author and year of publication | Article title | Eligible or not eligible? | Reason |
| --- | --- | --- | --- |
| Fernández-Sánchez et al. 2023 | Live birth rates following individualized dosing algorithm of follitropin delta in a long GnRH agonist protocol | Not eligible | Out of scope |
| Višnová et al. 2021 | Clinical outcomes of potential high responders after individualized FSH dosing based on anti-Müllerian hormone and body weight | Not eligible | Out of scope |
| Ishihara et al. 2022 | Comparison of ovarian response to follitropin delta in Japanese and White IVF/ICSI patients | Not eligible | Out of scope |
| Nyboe Andersen et al. 2017 | Individualized versus conventional ovarian stimulation for in vitro fertilization: a multicenter, randomized, controlled, assessor-blinded, phase 3 noninferiority trial | Eligible | - |
| Yang et al. 2022 | Comparative clinical outcome following individualized follitropin delta dosing in Chinese women undergoing ovarian stimulation for in vitro fertilization /intracytoplasmic sperm injection | Eligible | - |
| Qiao et al. 2021 | A randomised controlled trial to clinically validate follitropin delta in its individualised dosing regimen for ovarian stimulation in Asian IVF/ICSI patients | Eligible | - |
| Ishihara et al. 2021 | Randomized, assessor-blind, antimüllerian hormone-stratified, dose-response trial in Japanese in vitro fertilization/intracytoplasmic sperm injection patients undergoing controlled ovarian stimulation with follitropin delta | Eligible | - |
| Ishihara et al. 2021 | Individualized follitropin delta dosing reduces OHSS risk in Japanese IVF/ICSI patients: a randomized controlled trial | Eligible | - |
| Fernández-Sánchez et al. 2022 | A randomized, controlled, first-in-patient trial of choriogonadotropin beta added to follitropin delta in women undergoing ovarian stimulation in a long GnRH agonist protocol | Eligible | - |

**Final list of manuscripts included**

| First author and year of publication | Article title | Eligible or not eligible? | Reason |
| --- | --- | --- | --- |
| Nyboe Andersen et al. 2017 | Individualized versus conventional ovarian stimulation for in vitro fertilization: a multicenter, randomized, controlled, assessor-blinded, phase 3 noninferiority trial | Eligible | - |
| Yang et al. 2022 | Comparative clinical outcome following individualized follitropin delta dosing in Chinese women undergoing ovarian stimulation for in vitro fertilization /intracytoplasmic sperm injection | Eligible | - |
| Qiao et al. 2021 | A randomised controlled trial to clinically validate follitropin delta in its individualised dosing regimen for ovarian stimulation in Asian IVF/ICSI patients | Eligible | - |
| Ishihara et al. 2021 | Randomized, assessor-blind, antimüllerian hormone-stratified, dose-response trial in Japanese in vitro fertilization/intracytoplasmic sperm injection patients undergoing controlled ovarian stimulation with follitropin delta | Eligible | - |
| Ishihara et al. 2021 | Individualized follitropin delta dosing reduces OHSS risk in Japanese IVF/ICSI patients: a randomized controlled trial | Eligible | - |
| Fernández-Sánchez et al. 2022 | A randomized, controlled, first-in-patient trial of choriogonadotropin beta added to follitropin delta in women undergoing ovarian stimulation in a long GnRH agonist protocol | Eligible | - |
